# Closed Reduction and High-Strength Sutures for Transverse Patella Fractures: A Retrospective Analysis

**DOI:** 10.1007/s43465-023-00843-4

**Published:** 2023-02-27

**Authors:** Liben Huang, Xusong Li, Lin Ye, Shengsong Li

**Affiliations:** 1 Graduate School of Guangzhou University of Traditional Chinese Medicine, Guangzhou, Guangdong People’s Republic of China; 2 Zhongshan Hospital of Traditional Chinese Medicine, No. 3, Kangxin Road, West District, Zhongshan, 528401 Guangdong People’s Republic of China

**Keywords:** Patella fracture, Closed reduction, High-strength sutures, NICE knot

## Abstract

**Objective:**

This study aimed to investigate the clinical efficacy of closed reduction high-strength sutures combined with Nice knots in treating transverse patella fractures.

**Method:**

We retrospectively analyzed the clinical data of 28 patients who underwent surgery for transverse patella fractures from January 2019 to January 2020. Twelve cases of the study group were treated with closed reduction high-strength sutures combined with Nice knots, and 16 cases in the control group were treated with tension band wiring. Observations included patellar healing, follow-up knee mobility with Böstman score, Lysholm score, surgical data, postoperative complications, and secondary surgery rate.

**Result:**

No statistically significant difference was observed between the two groups in the Patient demographic data, with a mean follow-up of 13.14 ± 1.58 months. There was no delayed healing or deep infection in the two groups. In the control group, 2 cases of internal fixation failure and 1 case of superficial infection were observed. The differences in mean fracture healing time, follow-up Böstman score, Lysholm score and knee mobility between the two groups were not statistically different. However, the differences were statistically significant for the duration of surgery, Incision length, intraoperative bleeding and the secondary surgery rate was lower in the study group.

**Conclusion:**

Closed reduction high-strength sutures have good clinical efficacy in treating transverse patella fractures, with the advantages of shorter duration of surgery and Incision length,less intraoperative bleeding and no secondary removal.

## Introduction

The incidence of patellar fractures is approximately 1% [1] and approximately 70–90% are transverse fractures [2]. A patellar fracture directly affects the knee extension device; an uneven joint surface may lead to osteoarthritis[3]. Therefore, tension band wiring (TBW) is probably the most commonly used and effective surgical method[4, 5]. Still, inevitably, it has the disadvantages of protruding discomfort[6], soft tissue irritation[7], and the need for secondary surgical removal[8]. We used a closed repositioning method combined with high-strength sutures to overcome the problem of protruding implants without affecting stable fixation. We achieved remarkable results, which are summarized below.

## Materials and Methods

### General Information

After obtaining approval from the institutional ethical review committee, 28 patients with patella fracture 34-C1 admitted to the Department of Traumatic Orthopedics at our institution from January 2019 to January 2020 were retrospectively analyzed. Among them, 16 cases were in the Tension band wiring group; and 12 were in the high-strength sutures (HS) group.

Inclusion criteria: (1) closed transverse patellar fracture (AO/ATO 34 C1); (2) normal knee function before injury (3) age ≥ 18 years and ≤ 70 years. (4) Signed informed consent.

Exclusion criteria: (1) comminuted patellar fracture, (2) multiple fractures or injuries, (3) combined peripheral neurovascular injury, (4) inability to cooperate with follow-up or missing relevant information.

All patients provided written informed consent before the study. In addition, all patients were informed preoperatively about the minimally invasive treatment modality using closed reduction. They were duly informed about the possibility of using incisional reduction. Patients gave their informed consent and signed the informed consent form.

### Surgical Techniques

#### Preoperative Preparation

The patients were admitted to the hospital and externally immobilized with a plaster rest under extension on the same day. Preoperative prophylactic medication against VTE was routinely applied.

#### Anesthesia and Position

Subarachnoid block anesthesia or combined epidural anesthesia was selected, and the patients were placed in the supine position on the surgical bed.

#### Surgical Procedure

Combined lumbar and rigid or general anesthesia was chosen. Patients were placed in the supine position, and the part was disinfected and toweled.

#### HS Group

After repositioning the patella according to the patellofemoral joint surface closure, two scarf clamps or repositioning forceps were used on both sides of the patella to maintain the repositioning. Then two or three 1.5 or 2.0 mm clincher pins were placed percutaneously in the parallel longitudinal direction as temporary fixation. Next, two lateral and two medial incisions were made on each side of the patella at the superior and inferior patellar poles, respectively. Next, through four small incisions, the Ultrabraid NO.2 (Smith & Nephew, USA) was looped and "8" fixed, superiorly through the quadriceps; inferiorly through the inferior pole of the patella. Finally, the Nice knot was tied in the patellar epiphysis and embedded in soft tissue. Next, the kyphosis pin was pulled out in sequence, and the knot was then tied longitudinally through the bone tunnel through the skin and tightened using Nice. After bone fixation was completed, intraoperative fixation stability was assessed by flexing the knee to 90 degrees. The incision was finally sutured and bandaged.

#### TBW Group

A longitudinal incision was made in the anterior median of the knee, starting 2 cm proximal to the superior border of the patella and ending at the superior border of the tibial tuberosity. The skin and subcutaneous tissue were retracted to expose the patella. The fracture line was cleared of blood clots and embedded soft tissues. The wound and joint surface were thoroughly irrigated. The fracture block was fixed with a scarf clamp or repositioning forceps. The joint surface was flattened by lateral fluoroscopy with a C-arm machine. The superior and inferior fractures were fixed longitudinally with 1.5 or 2.0 mm diameter kerf pins through the patella. Then, 1 to 2, 0.8 mm diameter stainless steel sutures with stitches was tightened against the patella's superior and inferior pole to form an "8" tension band. After flexing the knee joint to confirm the fixation stability, the wound was irrigated and closed by layer, and drainage strips were placed.

### Postoperative Management

Patients in both groups received the same postoperative rehabilitation program. Patients performed active quadriceps, ankle, toe flexion, and extension functional exercises two days postoperatively. Afterward, patients were instructed to perform passive knee flexion and extension exercises post-surgery. Partial weight-bearing was acceptable starting on postoperative day 3. When the fracture was radiologically healed, full weight-bearing was allowed. Radiographs were reviewed 2, 4, 8, 12 weeks, and 6 months after surgery for fracture healing and to assess the function of the affected limb.

### Evaluation Index

Comparison of the duration of surgery, secondary surgery rate, incision length, complication rate, Böstman score [9], Lysholm score and knee mobility was performed between the two groups. Fracture healing time was determined by imaging data combined with clinical data.

### Statistical Analysis

SPSS 24.0 statistical software (IBM, Armonk, NY, USA) was used to statistically analyze the data. The measurement data were expressed as mean ± standard deviation to compare the demographic data (age, gender, type of injury and more), surgical data (duration of surgery, incision length, Intraoperative bleeding), and postoperative-related clinical data (complication rate, Böstman score, Lysholm score,and knee mobility). Categorical variables were expressed as frequencies or percentages, and continuous variables were expressed as mean ± SD. When the continuous variables were normally distributed, an independent sample t test was used to determine the differences between the two groups. Otherwise, nonparametric tests were performed for comparison. Categorical variables were compared using the Pearson chi-square test or Fisher's exact probability method. The postoperative Knee mobility and the functional knee scores were assessed using repeated measures analysis of variance in all statistical analyses. A value of *p* < 0.05 was considered statistically significant.

## Result

All 28 patients were healed till the last follow-up, with an average age of 56.32 ± 7.77 years and a follow-up of 13.14 ± 1.58 months. No significant difference was observed in patient demographic data between the two groups (Table 1).

**Table 1:** Patient demographic data and fracture characteristics

	TBW group	HS group	*P *value
Gender-*n* (%)			
Male	6(37.5)	5(41.67)	0.565
Female	10(62.5)	7(58.33)	
Age (y)	58.5 ± 7.74	53.41 ± 7.12	0.087
Follow-up time(mth)	13.19 ± 1.64	13.08 ± 1.56	0.867
Limb-*n* (%)			0.432
Left	12(75)	7(58.33)	
Right	4(25)	5(41.67)	
Mechanism-*n* (%)			0.560
Falling	1(6.25)	2(16.67)	
Bruise	15(93.75)	10(83.3)	
Smoking-*n* (%)			1
No	11(68.75)	8(66.67)	
Yes	5(31.25)	4(33.33)	
Diabetes-*n* (%)			0.624
No	14(87.5)	9(75)	
Yes	2(12.5)	3(25)	
Time from injury to operation (d)	3.87 ± 1.5	3.0 ± 1.2	0.103

### Comparison of Surgical Data, Complications, and Postoperative Function

The suture group outperformed the TBW group in terms of operative time (61.25 ± 9.80 vs. 76.56 ± 22.11, *p* = 0.022), incision length (3.08 ± 1.00 vs. 9.94 ± 1.24, *p* < 0.001), and secondary surgery rate (0 vs. 7, *p* = 0.024). The difference was statistically significant. (Table 2).

**Table 2:** Comparison of surgical data and complications

	TBW group	HS group	*P *value
Duration of surgery (min)	76.56 ± 22.11	61.25 ± 9.80	0.022
Incision length (cm)	9.94 ± 1.24	3.08 ± 1.00	0.000
Intraoperative bleeding (ml)	11.87 ± 11.67	4.58 ± 3.09	0.009
Secondary surgery (YES, %)	7(43.75)	0(0)	0.024
Complications			0.665
Superficial infection-*n* (%)	2(12.5)	0	
Failure of internal fixation-*n* (%)	1(6.25)	0	
Lower Extremity Vein Thrombosis-*n* (%)	2(12.5)	2(16.67)	
Healing time (mth)	3.56 ± 1.21	3.25 ± 0.87	0.444

**Fig. 1:**
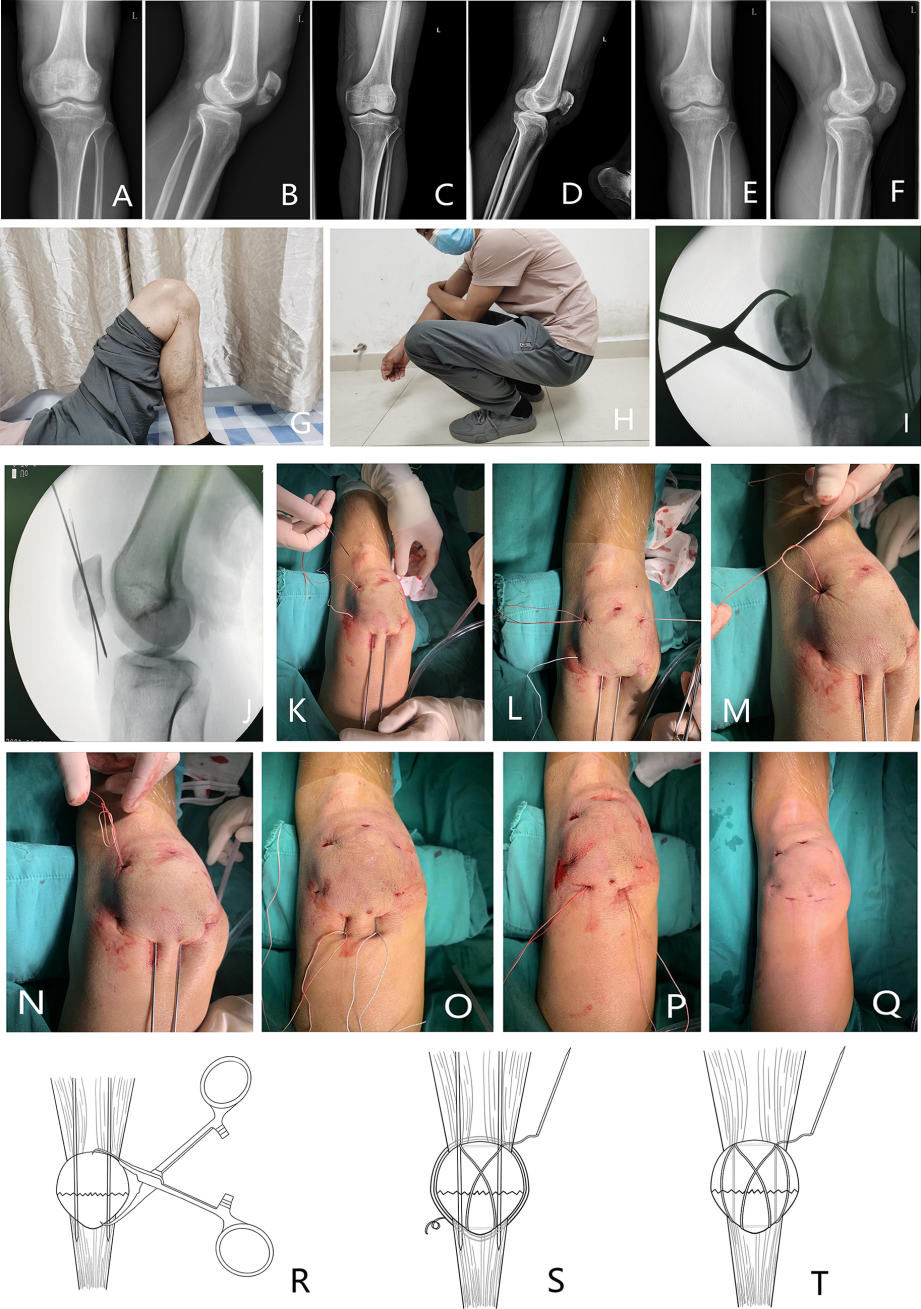
**A** and **B** Patient male, preoperative fluoroscopy of transverse patella fracture **C** and **D** postoperative fluoroscopy **E** and **F** 3 month postoperative fluoroscopic photo showing blurred fracture line. **G** and **H** postoperative 6-month functional photograph **I** and **J** intraoperative fluoroscopic photograph; **K**–**P**: The super-strength sutures were looped and fixed to the patella via the medial and lateral incisions, followed by "8" fixation and tensioning of the fracture end through the NICE knot. Next, the kerf pins were withdrawn, and the super sutures were fixed longitudinally through the bone tunnel with NICE knot fixation. **Q** Postoperative incision photograph. **R**–**T** Brief schematic diagram

Two cases (12.5%) of superficial infection and one case (6.25%) of internal fixation failure occurred in the TBW group, while none occurred in the super suture group. However, both groups detected lower extremity venous thrombosis in two cases Fig. 1. The difference in complications between the two groups was not statistically significant. (Table 2).

The results showed that the time effect was statistically significant (*p* < 0.001); No significant difference was observed in the impact of different treatment methods on functional recovery (*p* > 0.05). No significant difference was found in Knee mobility, Lysholm score, or Böstman score in the two groups at the 1-, 3-, 6-, or 12-month follow-ups (*P* > 0.05) (Table 3).

**Table 3:** Functional recovery

	TBW group	HS group	*P* value	95% CI
Böstman score Follow-up period				
1 month	11.25 ± 1.44	11.08 ± 1.56	0.772^*^	− 1.00–1.34
3 month	20.13 ± 2.06	20.83 ± 1.27	0.304^*^	− 2.09–0.68
6 month	24.5 ± 1.26	24.33 ± 1.30	0.736^*^	− 0.84–1.17
12 month	27.19 ± 1.51	27.75 ± 1.84	0.336^*^	− 1.74–0.62
Repeated measures analysis of variance			0.129^**^	
Lysholm score Follow-up period				
1 month	30.13 ± 2.13	28.67 ± 2.02	0.078^*^	− 0.17–3.09
3 month	46.5 ± 3.33	47.08 ± 2.02	0.149^*^	− 0.64–3.97
6 month	76.25 ± 2.18	75.42 ± 2.31	0.338^*^	− 0.92–2.59
12 month	88.44 ± 2.99	89.17 ± 2.89	0.523^*^	− 3.04–1.58
Repeated measures analysis of variance			0.105^**^	
Knee mobility (degree) Follow-up period				
1 month	107.31 ± 3.79	106.83 ± 3.83	0.743^*^	− 2.49–3.45
3 month	120.44 ± 3.50	121.17 ± 2.76	0.557^*^	− 3.25–1.79
6 month	124.18 ± 3.9	125.17 ± 4.26	0.533^*^	− 4.16–2.21
12 month	128.38 ± 5.46	129.83 ± 5.46	0.532^*^	− 6.18–3.27
Repeated measures analysis of variance			0.549^**^	

*CI* confidence interval

*Between-group *p* value tests for a significant between-group difference in the score at the time point

**Between-subject effects comparison

## Discussion

Recently, high-strength sutures have been used as a substitute for metallic materials and have achieved good clinical results. These results were obtained by Chen et al.[10] using braided polyester sutures (braided polyester) for patella fracture fixation. They conducted a matched historical control study and found no significant difference in healing and operative time compared to the wire tension band. However, the mean number of hospital days (4.04 ± 1.40 vs. 5.76 ± 1.50), number of procedures, and complication rate were significantly lower for nonmetallic fixation. We used the polyethylene braided material Ultrabraid NO.2 suture for internal fixation. The results of the mechanical comparison by Wüst et al. [11] showed that the difference in strength between Ultrabraid and FiberWire sutures was not significantly different without knots. In contrast, Ultrabraid (244 N) had better results with knots than FiberWire (189 N). Our study showed similar findings to these previous results. In our study, no significant difference was observed in healing time, final Böstman score, and knee mobility between the two groups compared to wire tension band wiring. While the differences in operation time, incision length, intraoperative bleeding, and secondary operation rate were statistically significant.

We used the NICE knot to maximize the tension of the seam, as the NICE knot is used in various applications [12]. *In-vitro* biomechanical analysis by Patel et al.[13] Showed the efficacy of non-metallic materials for the fixation of patella fractures comparable to other metallic materials using special knotting techniques. Further, Meyer et al. [14] tested 12 different knots and found that NICE knots had excellent mechanical strength. It provides a sliding locking feature besides performs well in preventing knot slippage. Thus, overcoming the difficulty of maintaining compression force in general surgical knots [15, 16].

Compared to the previous surgical approach, we used percutaneous sutures. Ma et al. [17] used percutaneous ring ligation to treat patellar fractures and obtained excellent reduction and functional results in 102 of 107 patients. Rathi et al. [18] treated 23 patients with transverse patellar fractures using a percutaneous keratomic pin tension band, where only 3 closed repositioning cases failed. The authors attribute this failure to the missed diagnosis of 2 cases of comminuted fractures and 1 case of small distal fracture. The remaining patients had radiographic healing at 8 weeks, and only one developed knee-femoral arthritis. Based on our previous experience, simple patella fractures can be treated by closed reduction. Therefore, a strict grasp of the fracture type may be the key to performing a good closed reduction. Furthermore, in cases where closed repositioning is not possible, a timely change to incisional repositioning is a wiser option. Although the differences in incision length and operative time were statistically significant in our study, the suture group (0 cases) performed better overall than the wire group (2 cases) in terms of postoperative infection control. However, the differences were not statistically significant, which may be attributed due to the small sample size.

Our study had some limitations. First, we conducted a retrospective cohort study with low evidence. Meanwhile, our small sample size and the short follow-up period lacked long-term efficacy evaluation. More high-quality, double-blind, randomized controlled trials with a larger number of cases are required to validate the advantages of the closed nonmetallic suture technique.
